# Prevention and Treatment of Peritoneal Dialysis-Associated Fibrosis with Intraperitoneal Anti-Fibrotic Therapy in Experimental Peritoneal Fibrosis

**DOI:** 10.3390/ph18020188

**Published:** 2025-01-30

**Authors:** Chiao-Yin Sun, Yu-Ting Hsieh, Shang-Chieh Lu, Chi-Ying F. Huang

**Affiliations:** 1 Department of Nephrology, Chang Gung Memorial Hospital, Keelung 61363, Taiwan; 2 Kidney Research Center and Department of Nephrology, Linkou Chang Gung Memorial Hospital, Taoyuan 333423, Taiwan; 3 Department of Medical Research, Chang Gung Memorial Hospital, Keelung 83301, Taiwan; yutinghsieh0913@gmail.com (Y.-T.H.); lsjstella0808@gmail.com (S.-C.L.); 4 Department of Biotechnology and Laboratory Science in Medicine, National Yang-Ming Chiao Tung University, Taipei 112304, Taiwan; 5 Institute of Biopharmaceutical Sciences, National Yang-Ming Chiao Tung University, Taipei 112304, Taiwan

**Keywords:** peritoneal dialysis, peritoneal fibrosis, anti-fibrotic therapy, nintedanib, pirfenidone

## Abstract

Background/Objectives: Long-term peritoneal dialysis (PD) often results in peritoneal damage and fibrosis, impairing peritoneal membrane function and leading to ultrafiltration failure. This study aimed to explore the therapeutic potential of nintedanib and pirfenidone in preventing and treating PD-associated peritoneal fibrosis using experimental models. Methods: An animal model of peritoneal fibrosis and cultured mesothelial cells were utilized to evaluate the effects of nintedanib and pirfenidone. Histological analysis, molecular techniques, and RNA sequencing were employed to assess the fibrosis, inflammation, and gene expression. The key outcomes included changes in the peritoneal structure, inflammatory markers, and transcriptional regulation. Results: Induced peritoneal fibrosis resulted in significant structural and histological changes. Treatment with nintedanib and pirfenidone effectively prevented peritoneal thickening and reduced excessive fibrosis deposition. Both agents ameliorated the inflammatory responses by lowering inflammatory marker expression, inhibiting cytokine activity, and decreasing macrophage infiltration. Molecular analyses revealed the suppression of inflammation-related transcription regulators and cytokine receptors. RNA sequencing identified glucose-induced gene expression changes and demonstrated significant modulation by the treatments. In animal studies with established fibrosis, these agents reduced peritoneal inflammation and slowed fibrosis progression. Conclusions: This study demonstrates that intraperitoneal administration of nintedanib and pirfenidone shows promise as an anti-fibrosis therapy for preventing and treating peritoneal fibrosis associated with PD. These findings highlight the potential of targeted interventions to improve the long-term outcomes for PD patients.

## 1. Introduction

Peritoneal dialysis (PD) serves as a common renal replacement therapy for patients with end-stage renal disease (ESRD). However, prolonged PD usage can induce peritoneal damage, leading to subsequent peritoneal fibrosis [1]. Peritoneal fibrosis, a prevalent morphological alteration seen in PD patients, may ultimately necessitate discontinuation of PD treatment. As peritoneal fibrosis advances, it compromises the peritoneal membrane’s function, resulting in ultrafiltration failure [2]. Additionally, peritoneal fibrosis significantly predisposes individuals to encapsulating peritoneal sclerosis, a severe complication of PD [3].

The progression of peritoneal fibrosis is intricate and involves multiple contributing factors. Factors such as the high glucose content in PD solutions, chronic inflammation, inflammatory cytokines, angiogenesis, and the mesothelial to mesenchymal transition contribute to its development [4]. The transformation of peritoneal mesothelial cells via the epithelial–mesenchymal transition, regulated by transforming growth factor-β (TGF-β), stands as a critical aspect of peritoneal fibrosis development. PD solutions containing high glucose levels and advanced glycation end-products can trigger the production of TGF-β [5]. Inflammatory pathways significantly contribute to peritoneal inflammation–fibrosis, with chronic inflammation mediated by immune cell infiltration and peritoneal angiogenesis playing pivotal roles in its progression [6,7].

To address peritoneal membrane fibrosis in PD patients, diverse strategies are under exploration. These encompass the utilization of biocompatible PD solutions, such as low-glucose and icodextrin-based solutions, alongside drug administration targeting anti-inflammatory and anti-fibrotic pathways involved in fibrosis development [1]. Pharmacological interventions aimed at mitigating matrix accumulation have proven beneficial in cellular and animal PD models. However, their usage in clinical studies for treating peritoneal fibrosis in PD patients remains contentious. While these solutions have demonstrated promise, their clinical benefits are still debated. Low-glucose solutions may not fully prevent fibrosis in the long term, and their cost is typically higher than that of conventional glucose-based solutions. Moreover, icodextrin has limited availability in some regions, and its effectiveness may vary between patients. Pharmacological treatments targeting anti-inflammatory and anti-fibrotic pathways, such as corticosteroids, immunosuppressants, and tamoxifen, have shown efficacy in preclinical models. They are capable of reducing matrix accumulation, fibroblast proliferation, and collagen deposition, which are hallmark features of fibrosis. Studies on corticosteroids and immunosuppressants for treating peritoneal fibrosis have failed to produce consistent results, and side effects like immunosuppression and infection risks limit their widespread use. Presently, there is insufficient evidence to recommend corticosteroids, immunosuppressants, or tamoxifen therapy for peritoneal dialysis-associated fibrosis [8,9].

Nintedanib and pirfenidone, both approved for clinical treatment of idiopathic pulmonary fibrosis, stand out as anti-fibrotic target therapies [10]. Nintedanib, a tyrosine kinase inhibitor, targets various growth factor receptors, like fibroblast growth factor receptor (FGFR), vascular endothelial growth factor receptor (VEGFR), platelet-derived growth factor receptor (PDGFR), colony-stimulating factor-1 receptor (CSF1R), and FMS-like tyrosine kinase 3 (FLT3), thus impeding fibroblast proliferation [11]. Conversely, pirfenidone disrupts TGF-β signaling and other growth factors, like PDGF and basic FGF, inhibiting fibroblast proliferation and collagen synthesis [12]. Both drugs have demonstrated anti-fibrotic and anti-inflammatory properties in in vitro and in vivo models of lung fibrosis [13].

Considering the involvement of multiple cytokines and growth factors in peritoneal fibrosis development, it is hypothesized that nintedanib and pirfenidone might offer therapeutic effects in preventing and treating peritoneal fibrosis associated with PD. By targeting multiple pathways involved in fibrosis—such as TGF-β signaling, fibroblast proliferation, and collagen deposition—these agents could offer more effective, multifaceted approaches to slowing or halting peritoneal fibrosis progression. Furthermore, the potential for intraperitoneal delivery of these drugs directly to the site of fibrosis could enhance their therapeutic effects while minimizing their systemic side effects. This targeted approach represents a significant innovation compared to traditional systemic therapies, which may not effectively reach the peritoneal membrane. To test this hypothesis, the effects of nintedanib and pirfenidone were investigated in experimental models of peritoneal fibrosis, both in vitro and in vivo. The results highlight the potential therapeutic effects of intraperitoneal anti-fibrotic target therapy with nintedanib and pirfenidone in preventing and treating PD-associated fibrosis.

## 2. Results

### 2.1. Intra-Peritoneum Anti-Fibrosis Target Therapy Prevents Peritoneum Fibrosis

**Figure 1:**
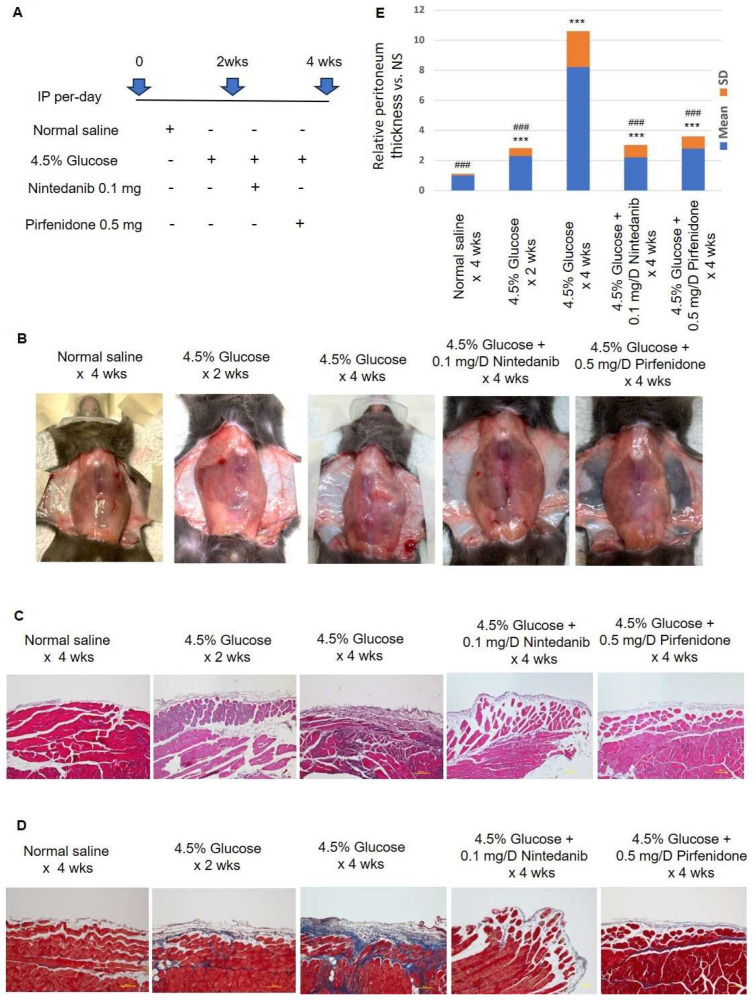
Macroscopic and microscopic peritoneum findings in the experimental peritoneum fibrosis animals. (**A**) Study design of the experimental peritoneum fibrosis. Animals that received daily normal saline IP injection were used as a control group. Experimental peritoneum fibrosis was induced by daily 4.5% glucose IP injection. Study groups received daily nintedanib (0.1 mg) or pirfenidone (0.5 mg) IP injection. (**B**) The representative macroscopic images of the peritoneum of experimental animals. (**C**) Results of the peritoneum hematoxylin and eosin staining. (**D**) Results of the peritoneum Masson’s trichrome staining. (**E**) Plots of the relative peritoneum thickness of the study animals (n = 10 for each group) (mean ± SD) (***: *p* < 0.001, vs. normal saline group) (^###^: *p* < 0.001, vs. 4.5% glucose group).

To investigate the therapeutic effects of nintedanib and pirfenidone in preventing peritoneal dialysis-associated peritoneum fibrosis, peritoneum fibrosis was induced by daily 4.5% glucose IP injection for 4 wks. In the treated model group, nintedanib and pirfenidone were injected intraperitoneally with glucose solution. The study protocol is demonstrated in Figure 1A. The gross peritoneum images of the study animals are showed in Figure 1B. The normal saline group showed a normal transparent peritoneal wall and sharply defined branching vessel structure in the parietal peritoneum. The 4.5% glucose groups (2 and 4 wks) showed a murky white color and an obscure branching vessel structure in the parietal peritoneum. Compared with the 4.5% glucose groups, the treatment groups, nintedanib and pirfenidone, had a slight white color and comparatively sharply defined branching vessels in the parietal peritoneum. The results of the peritoneum hematoxylin and eosin and Masson’s trichrome staining demonstrated that compared with normal mice, 4.5% glucose significantly induced peritoneum fibrosis. In contrast, nintedanib and pirfenidone treatment prevented peritoneal thickening and excessive fibrosis deposition (Figure 1C,D). The relative ratios of the thickness of the peritoneal membrane are plotted in Figure 1E.

### 2.2. Intra-Peritoneum Anti-Fibrosis Target Therapy Ameliorates Peritoneum Inflammation Reaction

**Figure 2:**
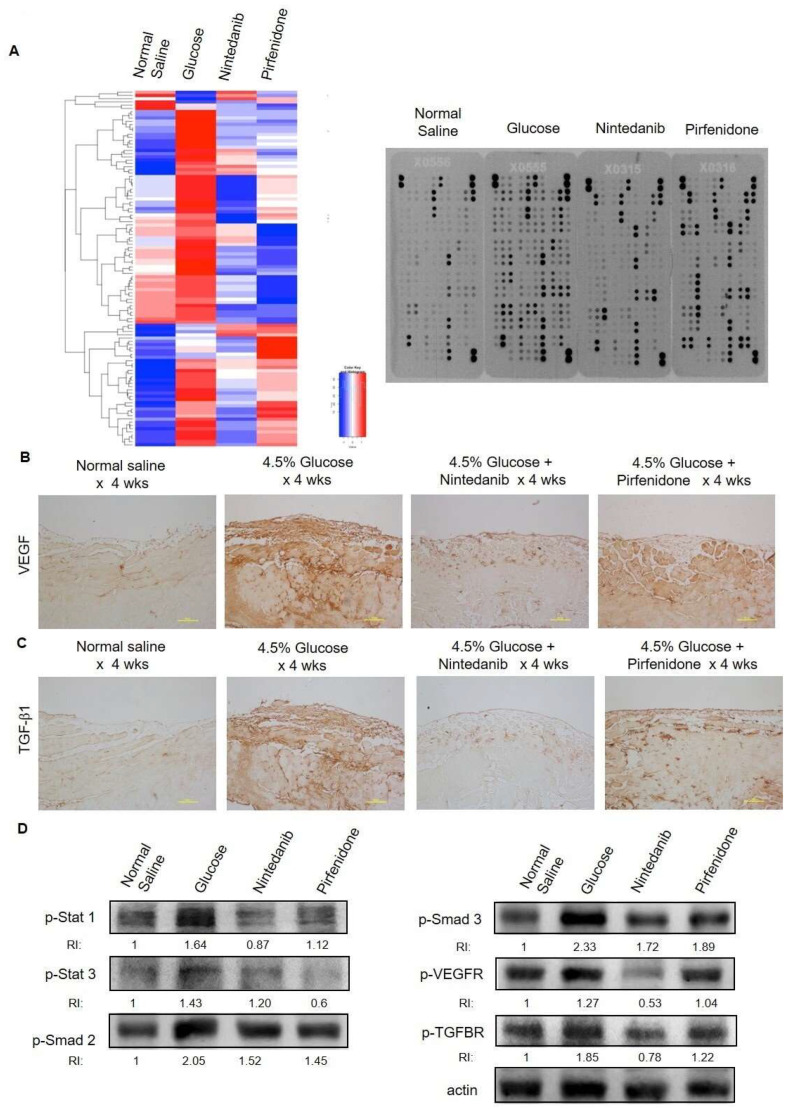
Peritoneal inflammatory reaction of experimental peritoneum fibrosis animals. (**A**) Results of the inflammatory cytokine array analysis. Peritoneum protein extracts (200 µg) of study animals were applied in this assay. The representative cytokine array films are presented. A heatmap of the mean spot pixel density of each study group was created. (**B**) Results of the immunohistochemistry staining for VEGF. (**C**) Results of the immunohistochemistry staining for TGF-ꞵ1. (**D**) Results of the Western blotting for inflammation-related transcription factors (p-Stat 1, p-Stat 3, p-Smad 2 and p-Smad 3) and receptors (p-VEGFR and p-TGFBR) (RI: relative intensity).

**Figure 3:**
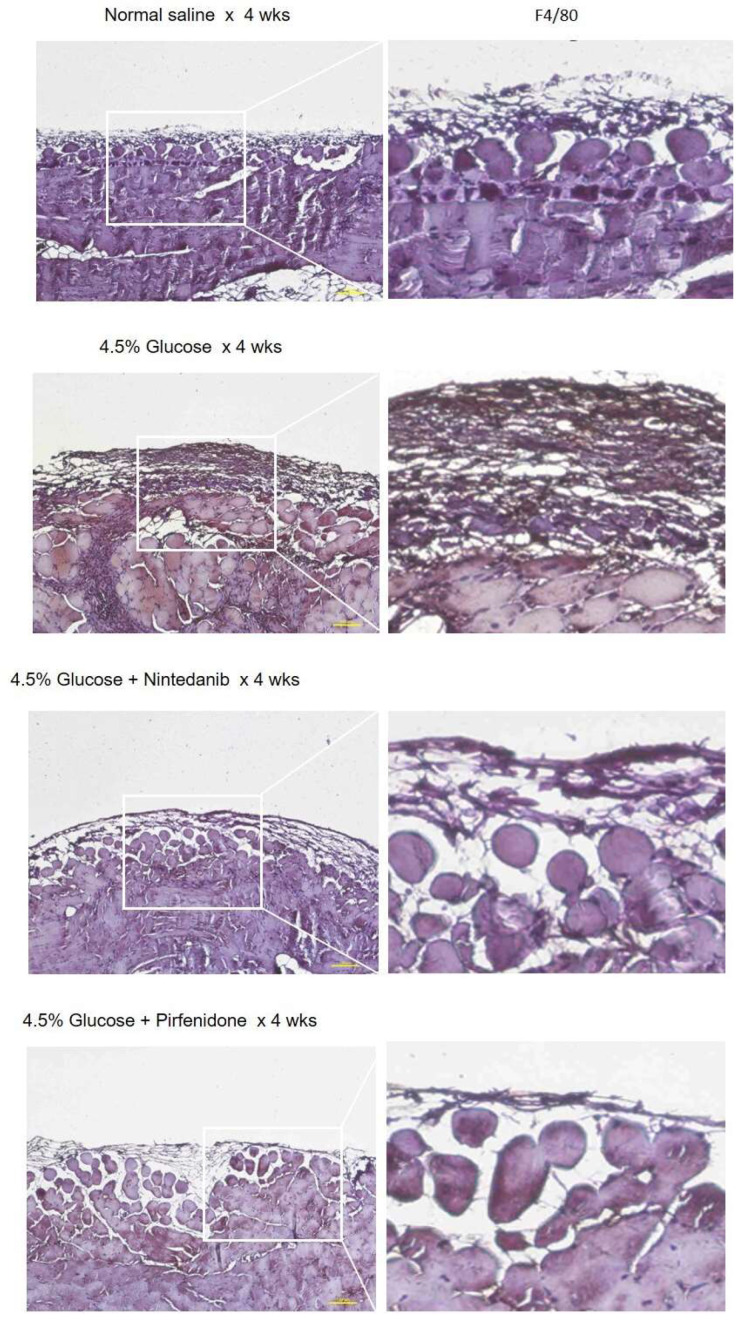
Peritoneum F4/80^+^ macrophage infiltration of experimental peritoneum fibrosis animals. Representative results of the immunohistochemistry staining for F4/80 with peritoneum tissue of study animals.

The protective effects of nintedanib and pirfenidone on peritoneal fibrosis were further demonstrated by the ability of ameliorating the peritoneum inflammation reaction. The results of the cytokine array analysis are shown in Figure 2A. A daily 4.5% glucose IP injection for 4 wks significantly induced peritoneum inflammation. It was found that nintedanib and pirfenidone treatment largely inhibited the peritoneal inflammation reaction by significantly reducing the expression of cytokines in the peritoneal tissues. The results of the immunohistochemistry staining also demonstrated that compared with the 4.5% glucose group, nintedanib and pirfenidone treatment significantly reduced the VEGF and TGF-ꞵ1 expression in the peritoneal tissues (Figure 2B,C). The Western blotting results showed that a daily injection of 4.5% glucose for 4 wks resulted in inflammation-related transcription regulators’ (Stat1, Stat3, Smad2 and Smad3) and cytokine receptors’ (VEGFR and TGFBR) activation. In the contrast, nintedanib and pirfenidone treatment significantly inhibited inflammation-related transcription regulators and cytokine receptors in the peritoneal tissues (Figure 2D). The anti-inflammation effect of nintedanib and pirfenidone was also evident, as shown by the peritoneum macrophage infiltration. The immunohistochemistry staining results demonstrated that compared with the 4.5% glucose group, nintedanib and pirfenidone treatment significantly alleviated peritoneum F4/80^+^ macrophage infiltration of the experimental animals (Figure 3).

**Figure 4:**
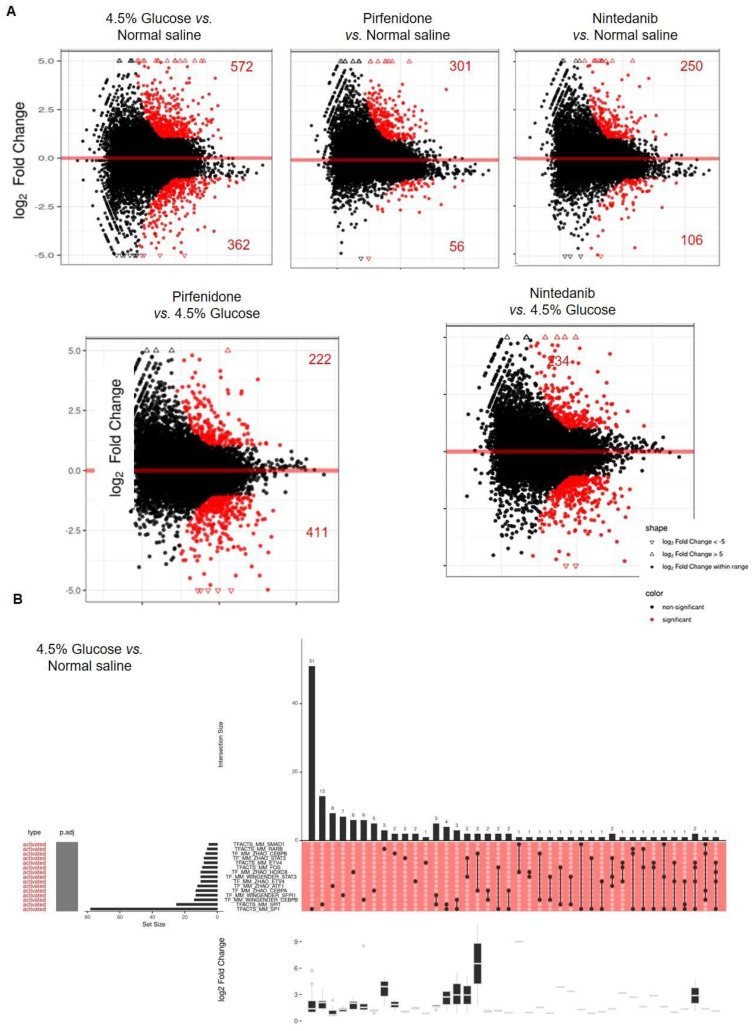

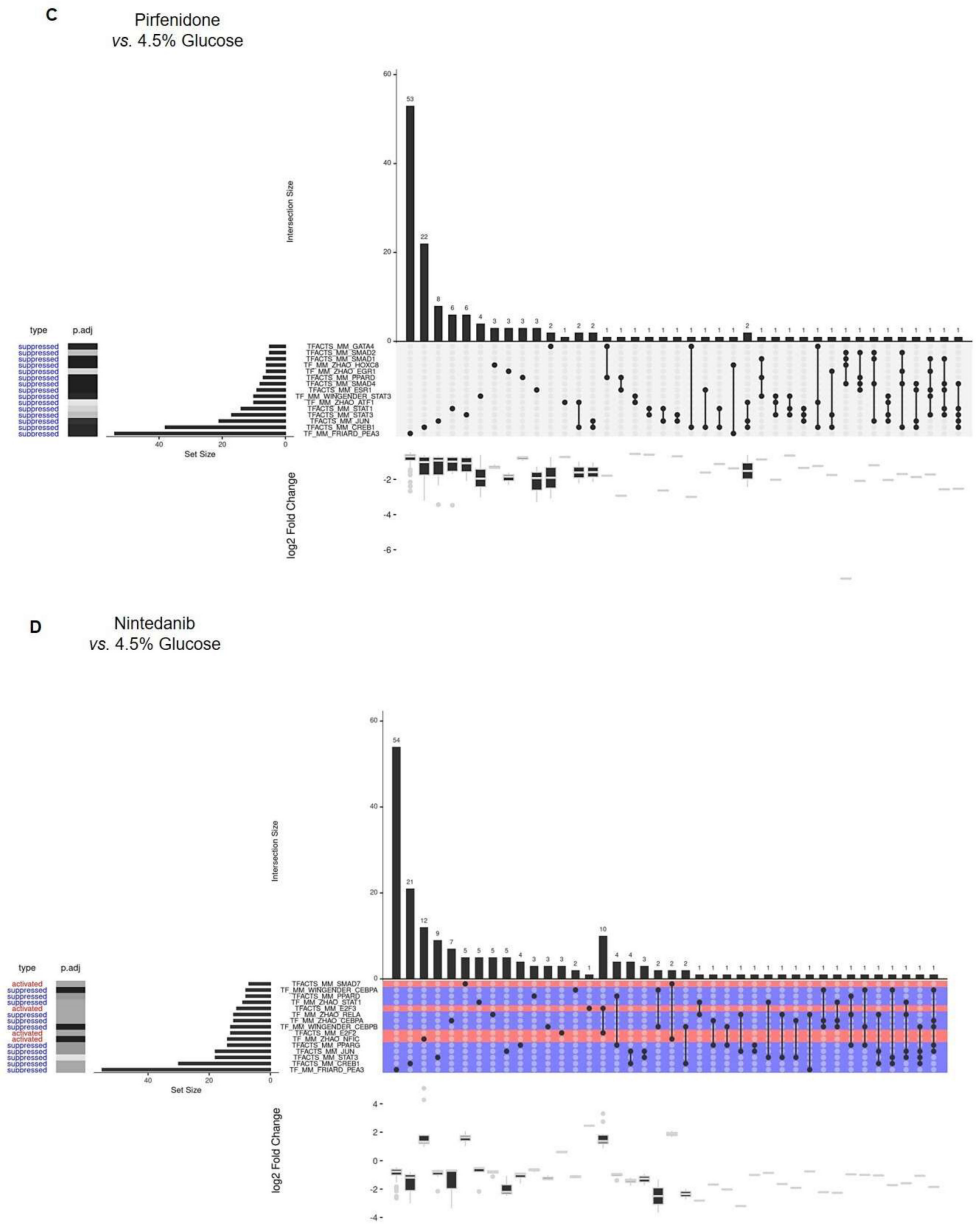
RNA sequencing results of the peritoneum of experimental peritoneum fibrosis animals. (**A**) Versus matrix MA plots. Numbers of up- and down-regulated genes are indicated in the figure legends. The comparison of samples from each group is presented in array format. The vertical axis is the log_2_ fold change, and the horizontal axis is the −log_10_ (pvalue/p.adjust). (**B**) Results of the GSEA, 4.5% glucose vs. normal saline group. (**C**) Results of the GSEA, pirfenidone vs. 4.5% glucose group. (**D**) Results of the GSEA, nintedanib vs. 4.5% glucose group. The right upper bar graph of the UpSet plot represents the size of the intersection. The central block from left to right is the enrichment category, p.adjust (the darker the color, the smaller the p value is and the more significant it is), and GeneSet size, name, and sample intersection type. The right lower box of the UpSet plot represents the range of the log_2_ fold change.

RNA sequencing analyses with peritoneum tissue were performed to investigate the global biological responses of the peritoneum using a whole-transcriptome analysis after exposure to 4.5% glucose. The versus matrix MA plots are showed in Figure 4A. Compared with the normal saline control group, 4.5% glucose exposure up-regulated 572 and down-regulated 362 genes. Nintedanib and pirfenidone treatment significantly reduced the genes up- and down-regulated by 4.5% glucose exposure (Figure 4A). The GSEA results demonstrated that 4.5% glucose exposure activated intrinsic apoptosis, regulation of transcription in response to stress, the response to unfolded protein and the autophagy signaling pathways (Figure 4B). Nintedanib and pirfenidone treatment significantly suppressed regulation of transcription in response to stress, the response to unfolded protein and the autophagy pathways, which were up-regulated by 4.5% glucose exposure (Figure 4A,C,D).

### 2.3. Anti-Fibrosis Target Therapy Reduces Apoptosis of Mesothelial Cells In Vitro

**Figure 5:**
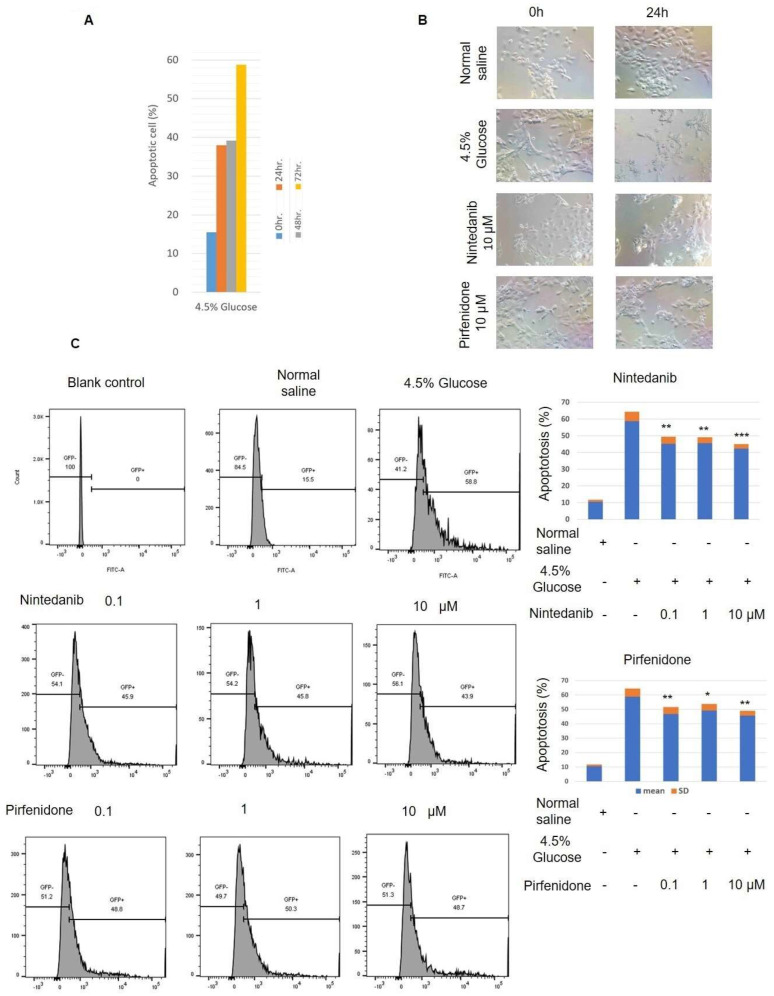
Here, 4.5% glucose induces apoptosis in cultured mesothelial cells. (**A**) TUNEL assay results of cultured mesothelial cells. Mesothelial cells were cultured in medium containing 4.5% glucose for the time indicated in the figure legend. The TUNEL assay was analyzed by flow cytometry. (**B**) Representative microscopy diagrams of cultured mesothelial cells. Mesothelial cells cultured in medium containing 4.5% glucose for 24 h were used for this study. (**C**) Representative flow cytometry of the TUNEL assay diagrams of cultured mesothelial cells. Cultured mesothelial cells treated with normal saline for 72 h were used as a control group. Treatment groups received 4.5% glucose and nintedanib or pirfenidone at concentrations of 0, 0.1, 1 and 10 μM. The percentage of apoptotic cells is plotted. Each study was repeated in triplicate (mean ± SD) (*: *p* < 0.05; **: *p* < 0.01; ***: *p* < 0.001 vs. 4.5% glucose).

**Figure 6:**
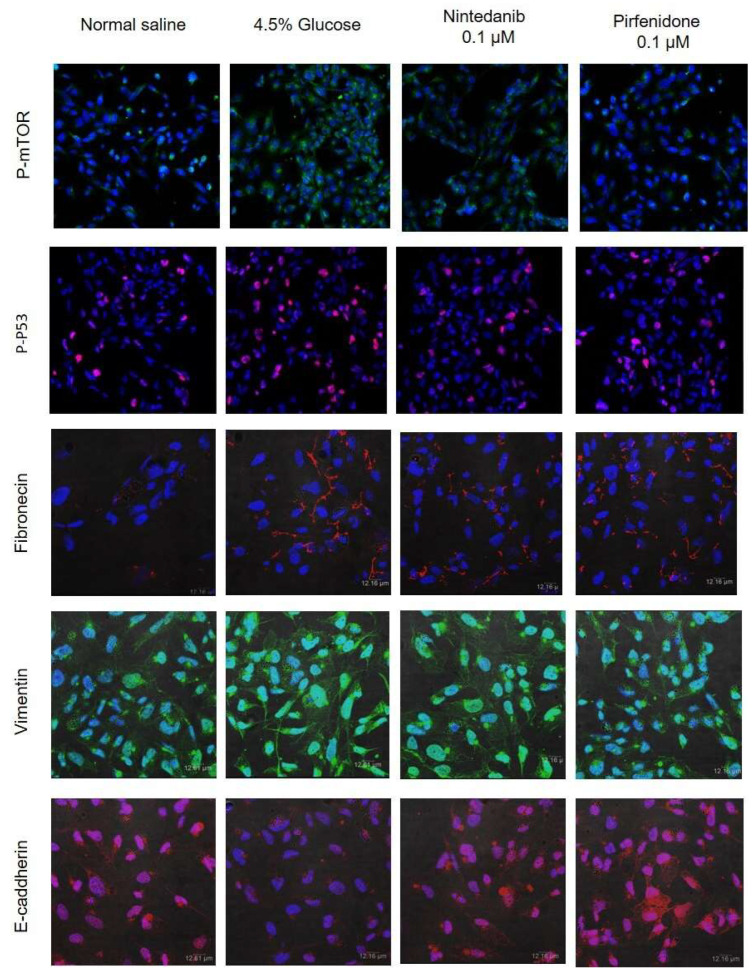
Immunofluorescence staining results of the epithelial–mesenchymal transition and apoptosis markers in cultured mesothelial cells. Representative fluorescence microscopy images of cultured mesothelial cells treated with medium containing 4.5% glucose for 24 h. The treatment studies were cultured with nintedanib or pirfenidone at concentrations of 0.1 μM for 24 h. The targets for the immunofluorescence staining are indicated in the figure legends.

The TUNEL assay results of the cultured mesothelial cells demonstrated that 4.5% glucose significantly induced mesothelial cell apoptosis. There was about 60% apoptosis of the cultured mesothelial cells after 72 h of 4.5% glucose treatment (Figure 5A). Microscopic studies showed that the cultured mesothelial cells had significant morphological deformity and decreased cellularity after 24 h of 4.5% glucose exposure. In contrast, nintedanib and pirfenidone treatment at a concentration of 10 μM significantly attenuated the morphological deformity and decreased the cellularity induced by glucose exposure (Figure 5B). The TUNEL assay results also revealed that nintedanib and pirfenidone treatment significantly reduced the apoptosis of the cultured mesothelial cells after 72 h of 4.5% glucose treatment (Figure 5C). Immunofluorescence staining also showed that 4.5% glucose treatment significantly increased the expression of apoptosis markers, p-mTOR and p-P53, in the cultured mesothelial cells. Nintedanib and pirfenidone treatment reduced the p-mTOR and p-P53 expression (Figure 6). In addition, the results of the fibronectin, vimentin and E-cadherin staining demonstrated that nintedanib and pirfenidone treatment reversed the epithelial–mesenchymal transition changes that were induced by 4.5% glucose exposure in cultured mesothelial cells (Figure 6).

### 2.4. Retarding Progressive Peritoneal Fibrosis in an Animal Model of Established Peritoneal Fibrosis

**Figure 7:**
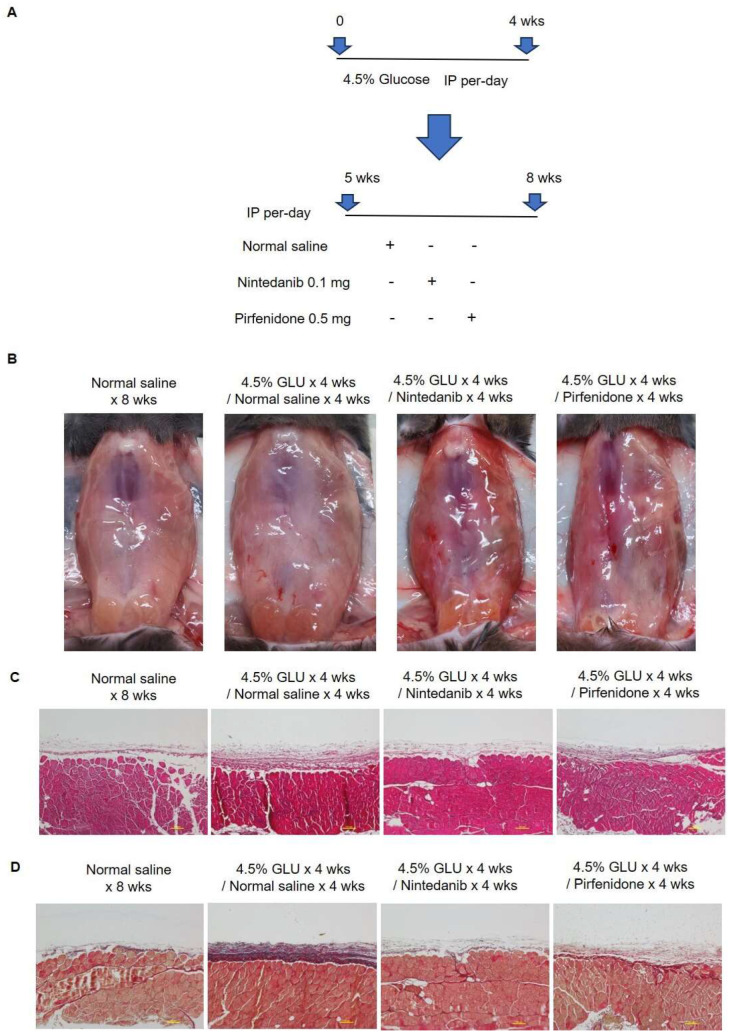
Treating peritoneum fibrosis in vivo. (**A**) Study design of the experimental peritoneum fibrosis. Animals received daily 4.5% glucose IP injection to induce peritoneum fibrosis. After 4.5% glucose injection for 4 wks, treatment studies were performed with daily normal saline, nintedanib (0.1 mg) and pirfenidone (0.5 mg) IP injection for another 4 wks. (**B**) The representative macroscopic images of the peritoneum of experimental animals. (**C**) Results of the peritoneum hematoxylin and eosin staining. (**D**) Results of the peritoneum Masson’s trichrome staining.

**Figure 8:**
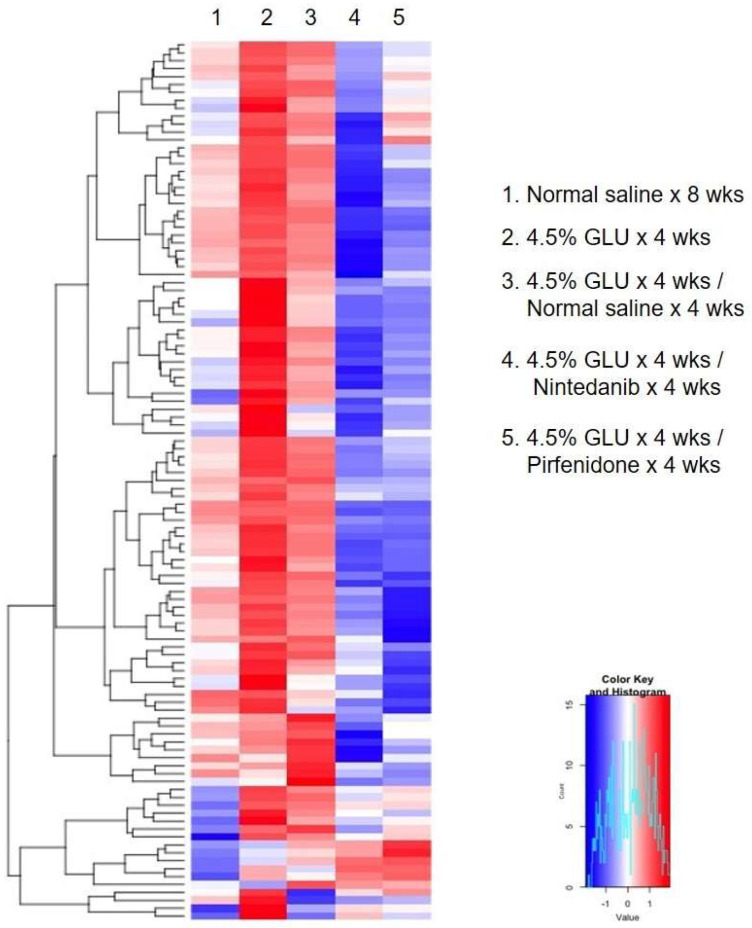
Peritoneal inflammatory reaction of experimental peritoneum fibrosis animals with intra-peritoneum anti-fibrosis treatment. Heatmaps of the cytokine array results were plotted. (1: normal saline × 8 wks; 2: 4.5% GLU × 4 wks; 3: 4.5% GLU × 4 wks/normal saline × 4 wks; 4: 4.5% GLU × 4 wks/nintedanib × 4 wks; 5: 4.5% GLU × 4 wks/pirfenidone × 4 wks).

To investigate the therapeutic effects of nintedanib and pirfenidone in treating established peritoneum fibrosis, mice with daily 4.5% glucose IP injection for 4 wks were used as an established peritoneal fibrosis model. The treatment studies included daily normal saline, nintedanib (0.1 mg) and pirfenidone (0.5 mg) injection for 4 wks (Figure 7A). The gross peritoneum images of the study animals are showed in Figure 7B. Compared with the normal saline treatment groups, the mice with nintedanib and pirfenidone treatment had comparatively transparent and defined branching vessels in the peritoneum. The results of the peritoneum hematoxylin and eosin and Masson’s trichrome staining demonstrated that compared with normal saline treatment, nintedanib and pirfenidone treatment significantly reduced the peritoneum thickness and retarded the peritoneal fibrosis (Figure 7C,D). It was also evident that nintedanib and pirfenidone treatment largely inhibited the peritoneal inflammation reaction by significantly reducing the expression of cytokines in the established peritoneal fibrosis model (Figure 8).

## 3. Discussion

Peritoneal membrane damage leading to PD failure remains a primary constraint in long-term PD therapy [14]. Local peritoneal inflammation is the driving force behind this damage, causing structural alterations like fibrosis, marked by sub-mesothelial compact zone thickening and vascular damage [15,16]. These alterations affect solute transport across the membrane, resulting in dialysis failure [17,18].

This study showcased the preventive effects of intraperitoneal anti-fibrosis therapy using nintedanib and pirfenidone against peritoneal fibrosis induced by daily 4.5% glucose injections over 4 weeks in an experimental model. The therapy not only ameliorated inflammation by reducing cytokine expression and inhibiting inflammation-related pathways but also countered the glucose-induced genetic alterations associated with the stress response and apoptosis, as highlighted by RNA sequencing. In vitro studies further confirmed the ability of nintedanib and pirfenidone to reduce the mesothelial cell apoptosis induced by glucose exposure and reverse the epithelial–mesenchymal transition changes. Notably, when administered after fibrosis establishment, the therapy exhibited potential therapeutic effects by reducing fibrosis and suppressing inflammation.

Pirfenidone, an anti-inflammatory and anti-fibrotic drug, inhibits collagen synthesis and fibroblast proliferation [19]. Nintedanib, a tyrosine kinase inhibitor targeting growth factor pathways [20], has shown therapeutic potential beyond idiopathic pulmonary fibrosis, including in diseases like systemic sclerosis and chronic kidney disease [21,22]. Intraperitoneal delivery offers a localized therapeutic approach, enhancing drug bioavailability at the target site while minimizing systemic exposure. This method’s efficacy has been demonstrated in PD peritonitis studies, where intraperitoneal antibiotics outperformed intravenous administration [23]. Previous experimental models of peritoneal fibrosis suggested the potential therapeutic effects of orally administered nintedanib [24,25]. However, clinical evidence indicated possible side effects associated with systemic administration of both therapies [26,27]. Considering the PD peritonitis studies, intraperitoneal antibiotics were more effective than intravenous antibiotics in reducing treatment failure [28,29], suggesting that oral administration of nintedanib and pirfenidone might not be optimal. This study’s focus on intra-peritoneum anti-fibrosis therapy, specifically using nintedanib and pirfenidone, presents compelling evidence of their potential in preventing and alleviating peritoneal fibrosis linked to PD.

Inflammation, especially its association with the TGF-β pathway, significantly contributes to peritoneal fibrosis’s development and progression. TGF-β, a key mediator in driving fibrotic changes, is up-regulated in response to inflammatory triggers like exposure to glucose solutions in peritoneal dialysis [30]. Targeting TGF-β signaling, as achieved by nintedanib and pirfenidone, has shown efficacy in experimental models by modulating its expression and downstream effects, reducing the fibrotic changes [31,32]. Understanding the interplay between inflammation, TGF-β, and peritoneal fibrosis is crucial for developing targeted therapies to disrupt the cascade of events leading to fibrosis. Addressing inflammation and targeting specific pathways like TGF-β might mitigate or prevent peritoneal fibrosis development and progression. The results demonstrated that nintedanib and pirfenidone significantly inhibited TGF-β signaling induced by high glucose concentrations. Even in established peritoneal fibrosis, delayed administration of these drugs could inhibit TGF-β signaling activation.

## 4. Materials and Methods

### 4.1. Experimental Peritoneum Fibrosis Animal Model

Ten-week-old B6 mice were utilized in this study, categorized into the control, peritoneal fibrosis, and peritonitis models. The control group received daily intraperitoneal (IP) injections of normal saline (3 mL). The peritoneal fibrosis group received daily IP injections of 4.5% glucose dialysate (3 mL) [33]. The treatment groups received nintedanib (0.1 mg) (SML2848, Sigma-Aldrich, St. Louis, MO, USA) or pirfenidone (0.5 mg) (P2116, Sigma-Aldrich) dissolved in 10 uL DMSO, along with daily IP injections of 3 mL 4.5% glucose solution or normal saline [34,35]. This study’s flow and treatment course are depicted in the figure legends (n = 10 for each study group). The sample sizes were based on previous experimental studies and the relevant literature, where similar group sizes were shown to achieve adequate statistical power in comparable animal models of peritoneal fibrosis [33]. Parietal peritoneum tissues, excluding the injection site, were harvested under anesthesia. All the animal procedures adhered to the guidelines of the Institutional Animal Care and Use Committee at Chang Gung Memorial Hospital, Keelung, Taiwan.

### 4.2. Histopathological Examination

Sections of paraffin-embedded specimens underwent staining with hematoxylin and eosin, and with Masson’s trichrome, observed under a high-power field (×200) to evaluate the peritoneal thickness and fibrosis. The staining procedures followed the protocol provided by the manufacturer (Sigma-Aldrich). The peritoneal membrane thickness was assessed by measuring the distance from the mesothelium surface to the upper limit of the muscular tissue at 10 random points, comparing the mean thickness of each tissue sample [36].

### 4.3. Cytokine Array Analysis

Peritoneum protein lysates were utilized for the multiplex cytokine array analysis (Proteome Profiler Mouse Cytokine Array Kit, R & D). Here, 200 µg of lysate per array was used, and the analysis followed the manufacturer’s protocol (R & D). Array images were collected and analyzed using the LI-COR Odyssey Infrared Imaging System (LI-COR, Inc.). The mean spot pixel density was calculated using image analysis software (ImageJ, National Institutes of Health). The cytokine array list is included in Figure S1.

### 4.4. Immunohistochemistry and Immunofluorescent Staining

The parafilm peritoneum samples were cut into 4 µm sections. The hydrated deparaffinization tissues were incubated with the primary antibodies at 37 °C for 2 h, then incubated with a horseradish peroxidase secondary antibody (Sigma-Aldrich) at 37 °C for 1 h. The primary antibodies for the immunohistochemistry staining and the folds of dilution are described as follows: VEGF (Cell Signaling, 500×); TGF-ꞵ1 (Cell Signaling, 500×); and F4/80 (Cell Signaling, 500×).

Cryostat sections of cultured mesothelial cells fixed with ice-cold acetone were incubated overnight at 4 °C with primary antibodies against mouse; this was followed by incubation with appropriate secondary immunofluorescent antibodies for 1 h at room temperature. The antibodies for the immunofluorescence staining and the folds of dilution are described as follows: phosphorylated mammalian target of rapamycin (p-mTOR) (Cell Signaling, 500×); phospho-p53 (p-P53) (Cell Signaling, 500×); fibronectin (Abcam, 500×); vimentin (Cell Signaling, 100×); and E-cadherin (Cell Signaling, 100×). The cell nucleus was stained with Hoechst stains (Sigma-Aldrich). The stained samples were observed under a confocal microscope (Leica Microsystems, Wetzlar, Germany).

### 4.5. Western Blotting

Peritoneum tissues were homogenized and the total protein was extracted using a commercial kit as per the manufacturer’s instructions (Protein Extraction Kit, Millipore, St. Louis, Mo, USA). Protein extracts (30 μg protein per lane) were mixed with a sample loading buffer and separated on 12% sodium dodecyl sulfate polyacrylamide gel. The proteins were electro transferred onto polyvinylidene fluoride membranes (0.2 μm: Immun-Blot, Bio-Rad (Hercules, CA, USA)). The antibodies used for the Western blotting are listed as follows: phosphorylated Stat-1(p-Stat 1)/p-Stat 3/phosphorylated Smad 2 (p-Smad 2)/p-Smad 3 (Abcam, 500×); p-VEGFR/p-TGFBR (Cell Signaling, 500×); and β-actin (Abcam, 1000×). The intensity of each band was quantified using the NIH Image software, (version 1.49), and the densitometric intensity corresponding to each band was normalized against the β-actin expression.

### 4.6. RNA Sequencing

The total RNA of the peritoneum tissues was extracted by using a commercial kit (RNeasy Kit, Qiagen, Germantown, MD, USA) according to the manufacturer’s instructions, including DNase treatment. The total RNA (10 μg) was then reverse transcribed by reverse transcriptase (Bio-Rad) with random primers. Following quality control, the RNA was enriched using oligo(dT) beads. The cDNA library concentration was quantified with a Qubit 2.0 fluorometer (Life Technologies,Carlsbad, CA, USA). Subsequently, it was diluted to 1 ng/µL before assessing the insert size on an Agilent 2100 and further quantification for accuracy using quantitative polymerase chain reaction (ensuring library activity >2 nM). Finally, the libraries were fed into Illumina machines based on the activity and expected data volume. The data analysis involved aligning it with a reference genome for interpretation. Differentially expressed genes (DEGs) analysis of two conditions was performed in R using DEGseq2 [37]. GO and KEGG pathway enrichment analyses of the DEGs were conducted [38]. The resulting *p*-values were adjusted using Benjamini and Hochberg’s approach for controlling the FDR. The analysis step first sorted the RNA-Seq data according to the expression change (Fold Change), then marked the genes in the target defined gene set on the sorted gene list, and finally, calculated the enrichment score (ES) according to the gene order. The calculation method for the ES used random walk to scan the sorted gene list from scratch. Gene set enrichment analysis (GSEA) was performed with 1000 permutations to identify enriched biological functions and activated pathways from the molecular signatures database (MSigDB, v7.2) [39].

### 4.7. Mesothelial Cell Culture, TUNNEL Assay and Flow Cytometry

The mesothelial cell line, MeT-5A, was obtained from ATCC and cultured in a condition according to the ATCC’s recommendation [40]. Cultured mesothelial cells were used for the terminal deoxynucleotidyl transferase dUTP nick end labeling (TUNEL) assay. TUNEL staining was performed using a MEBSTAIN Apoptosis Kit Direct (MBL International, Woburn, MA, USA) according to the manufacturer’s protocol. Cultured cells with TUNEL staining in the FACS buffer were analyzed and sorted with BD FACSAria III (Becton, Dickinson and Company, Franklin Lakes, NJ, USA)

### 4.8. Statistical Analysis

All the data were initially assessed for normality using the Shapiro–Wilk test. The data were determined to follow a normal distribution; therefore, the results were expressed as the mean ± SD. The homogeneity of variance was evaluated using Levene’s test. A one-way ANOVA was applied as the primary analysis. The post hoc analysis was performed using Tukey’s test. *p*-values of <0.05 were considered statistically significant.

## 5. Conclusions

This study underscores the potential of intraperitoneal nintedanib and pirfenidone as effective strategies for preventing and treating peritoneal fibrosis associated with PD. By targeting inflammation, TGF-β signaling, and fibrotic changes, these therapies demonstrate significant efficacy in both preventative and therapeutic settings. Their ability to mitigate established fibrosis further highlights their clinical potential. The potential synergistic effects of combining nintedanib and pirfenidone with other anti-fibrotic or anti-inflammatory agents should be investigated.

## Data Availability

The raw RNA-seq data generated in this study are available from the corresponding author upon request. Data will be made available on request.

## Supplementary Materials

The following supporting information can be downloaded at https://www.mdpi.com/article/10.3390/ph18020188/s1, Figure S1: List of cytokine array.
