# Supplementary figures and images for: Prevention and Treatment of Peritoneal Dialysis-Associated Fibrosis with Intraperitoneal Anti-Fibrotic Therapy in Experimental Peritoneal Fibrosis
Source: Pharmaceuticals (Basel). 2025 Jan 30;18(2):188. doi: 10.3390/ph18020188 (PMC11859390; doi:10.3390/ph18020188)

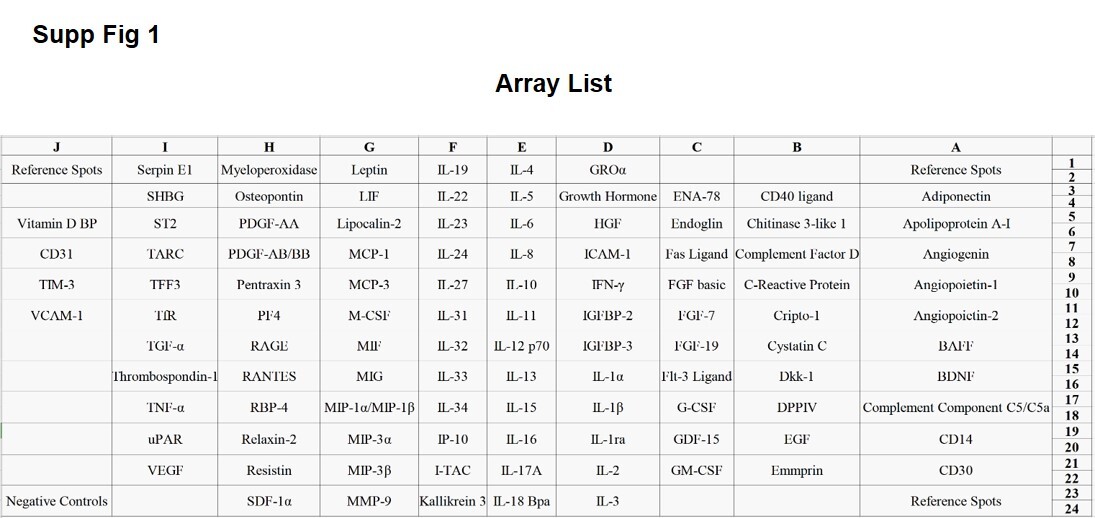

Supplement: Supplementary file 1 [file pharmaceuticals-18-00188-s001.zip › pharmaceuticals-3357640-supplementary.jpg]
